# Serum Apolipoproteins C-I and C-III Are Reduced in Stomach Cancer Patients: Results from MALDI-Based Peptidome and Immuno-Based Clinical Assays

**DOI:** 10.1371/journal.pone.0014540

**Published:** 2011-01-18

**Authors:** Meital Cohen, Rami Yossef, Tamir Erez, Aleksandra Kugel, Michael Welt, Mark M. Karpasas, Jonathan Bones, Pauline M. Rudd, Julien Taieb, Herve Boissin, Dror Harats, Karin Noy, Yoram Tekoah, Rachel G. Lichtenstein, Eitan Rubin, Angel Porgador

**Affiliations:** 1 The Shraga Segal Department of Microbiology and Immunology and the National Institute for Biotechnology in the Negev, Ben Gurion University of the Negev, Beer Sheva, Israel; 2 Analytical Research Services & Instrumentation Unit, Ben Gurion University of the Negev, Beer Sheva, Israel; 3 Oxford Glycobiology Laboratory, The National Institute for Bioprocessing Research & Training (NIBRT), Conway Institute, University College Dublin, Dublin, Ireland; 4 RNTech SAS France, Paris, France; 5 The Avram and Stella Goren-Goldstein Department of Biotechnology Engineering, Ben Gurion University of the Negev, Beer Sheva, Israel; 6 The Bert Stassburger Lipid Center, Sheba Medical Center, Ramat Gan, Israel; 7 The Life Sciences Department, Ben Gurion University of the Negev, Beer Sheva, Israel; Technische Universität München, Germany

## Abstract

Finding new peptide biomarkers for stomach cancer in human sera that can be implemented into a clinically practicable prediction method for monitoring of stomach cancer. We studied the serum peptidome from two different biorepositories. We first employed a C8-reverse phase liquid chromatography approach for sample purification, followed by mass-spectrometry analysis. These were applied onto serum samples from cancer-free controls and stomach cancer patients at various clinical stages. We then created a bioinformatics analysis pipeline and identified peptide signature discriminating stomach adenocarcinoma patients from cancer-free controls. Matrix Assisted Laser Desorption/Ionization–Time of Flight (MALDI-TOF) results from 103 samples revealed 9 signature peptides; with prediction accuracy of 89% in the training set and 88% in the validation set. Three of the discriminating peptides discovered were fragments of Apolipoproteins C-I and C-III (apoC-I and C-III); we further quantified their serum levels, as well as CA19-9 and CRP, employing quantitative commercial-clinical assays in 142 samples. ApoC-I and apoC-III quantitative results correlated with the MS results. We then employed apoB-100-normalized apoC-I and apoC-III, CA19-9 and CRP levels to generate rules set for stomach cancer prediction. For training, we used sera from one repository, and for validation, we used sera from the second repository. Prediction accuracies of 88.4% and 74.4% were obtained in the training and validation sets, respectively. Serum levels of apoC-I and apoC-III combined with other clinical parameters can serve as a basis for the formulation of a diagnostic score for stomach cancer patients.

## Introduction

Mortality rates of many cancers have not changed dramatically in the last 20 years [1]. Early detection was shown to greatly improve the efficacy of cancer treatment, yet detection is often only possible after the appearance of the first clinical symptoms, which in some cancers occurs too late for successful intervention. This is largely due to the absence of specific and sensitive tests that allow early screening and monitoring of cancerous states. Therefore, the discovery of novel tumor biomarkers is increasingly considered critical to improving cancer treatment. In the past decade, many studies have focused on biomarker discovery. One of the most promising sources for biomarker discovery is the human blood, in particular serum and plasma, which can reflect many events in the body, in real time. Yet, despite immense efforts, only a very small number of plasma proteins have been proven to have diagnostic value [2]–[5]. Frequently, these biomarkers do not stand alone and are accompanied by other tests for monitoring and diagnosis. Most of them are not specific and sensitive enough for wide screen diagnosis [6], [7].

One possible source of novel cancer biomarkers is the peptidome. The rationale behind focusing on serum peptides is based on evidence that cancer formation and development involves change in proteins' and peptides' metabolism, and on the increased availability of methodology for screening the entire peptidome. In terms of cancer development, changes may occur in the array of intra- and extra-cellular peptides represented in the blood peptidome, which may be specific to the cancerous stage, and thus have a diagnostic potential [2], [4], [5]. In terms of detection technology, recent advances in MS technology enable the detection of hundreds of peptides from a few microliters of serum [8], [9]. Indeed, previous blood peptidome studies reported an array of signature peptides in serum that had distinguished healthy from cancer patients (reviewed in [5]). This was shown for prostate, bladder, breast and thyroid cancer by Villanueva *et al* [10], [11]. They reported 61 signature peptides that could distinguish healthy individuals from 3 different types of cancer patients. While all of these peptides and/or their fragments are normally found in the serum, differences in quantity between healthy and affected individuals are observed. However, although these results demonstrate the potential that peptidome profiles have for cancer diagnosis, it still remains to be shown that this approach can be extended to discover biomarkers suitable for early diagnosis and consistent monitoring. First, the ability of these sera peptide biomarkers to distinguish patients from controls was mostly demonstrated for patients with highly advanced or metastatic tumors. Moreover, the robustness of these biomarkers has been challenged; uncontrolled variables, mostly attributed to differences in sample handling, processing protocols and data analysis, have been shown to dramatically modify the results of these assays [11]–[19]. By putting major emphasis on sample acquisition, handling, processing, MS signal processing and statistical analyses more robust and reproducible results can be achieved [18], [20], [21].

In this work, we focused on discovering an array of signature peptides that could have diagnostic value for stomach cancer. In order to achieve this, we used three different serum sources involving stomach cancer patients at different stages. A strict protocol for serum collection and processing was applied [18], using a cohesive procedure of peptide extraction and MALDI-TOF readings, with a modified analysis pipeline. Together, the improved pipeline allowed for the identification of a peptide pattern that discriminates between cancer and control samples. These results were corroborated on the original and new sera for three identified features from the pattern, apoC-I (two features) and apoC-III, using immuno-based assays. We then employed serum levels of apoC-I and apoC-III combined with CRP and CA19-9 markers to discriminate stomach adenocarcinoma patients from cancer-free controls.

## Materials and Methods

### Serum harvesting and handling

Sera were obtained from two commercial sources. 79 sera samples from pre-operation stomach cancer patients and 33 sera samples from cancer-free matched controls (including 10 gastritis patients) were collected by RNTech (Paris, France) in Romania. Sera form cancer and non cancer patients were taken after overnight fasting in the following manner: 5 ml of blood was drawn into a vacuette serum tube (Cat #456005, Greiner Bio One, Kremsmuenster, Austria) and left to clot for about 30 minutes, after which the tube was centrifuged at 3,000 rpm on a Hettich EBA 20S centrifuge (Hettich Ag, Tuttlingen, Germany) for 5 minutes at room temperature. The separated serum was aliquoted into 1 ml aliquots in sterile cryogenic tubes (Nalgene, Rochester, NY, USA) and immediately frozen at (−70)°C. 22 pre-operation stomach cancer sera and 21 controls were collected by Asterand in the USA (Detroit, MI, USA) in the following manner: 10 ml of blood was drawn into a BD vacutainer SST plus plastic tube (cat #BEC 367985, BD, San Jose, CA, USA). The tube was mixed by inverting it 5 times and left to clot for about 30 minutes in a vertical position. This step was followed by a centrifugation of 1,100–1,300 g for 10 minutes at room temperature. The separated serum was aliquoted into 1 ml aliquots in sterile cryogenic tubes (Nalgene) tubes and immediately frozen at (−70)°C. For the Asterand source, fasting data was not collected on any of the blood draws in their bank. Sera samples from both companies were transported on dry ice and stored at (−70)°C immediately upon arrival. Sera samples were thawed on ice for about an hour and a half, 50 µl was aliquoted into lo-bind tubes (Eppendorf, Hamburg, Germany) and immediately re-frozen at (−70)°C. All sample aliquots were stored at (−70)°C until processing. A third source of sera was obtained in our laboratory from 12 cancer-free Israeli controls. Blood was drawn with the tube brand used by RNTech (Cat #456005, Greiner Bio One) and serum handling followed the procedure of RNTech. The sera obtained in our laboratory were taken from non-fasting individuals. Both RNTech and Asterand companies have established and conducted their activities following regulatory and ethical standards, implementing local, national, European, US and International (UN) rules and recommendations particularly when applicable to biological material collection and treatment and research result exploitation. This includes both written consent of each patient contributing to the biological and data bank, and written study authorization from the ethics committee of each clinical institute contributing samples to the companies' banks.

### Serum sample processing and preparation for MS-MALDI reading

Each serum sample was processed in two to three replicates (from identical aliquots and on separate random dates). Peptides were extracted on beads coated with C8, washed, eluted, mixed with CHCA matrix, and deposited on the MALDI target plate. Sera were processed in replicates and deposited onto the MALDI plate in duplicates. For detailed description please see File S1.

### Data analysis of MALDI results

Data processing was performed in two steps. In the first step, an intensity matrix was performed from the raw ASCII files of MALDI-TOF readings from all sera sample sources using re-sampling, aligning, and m/z peaks detection as described in Villanueva *et al* [21]. In the second step, machine learning was used to define a discriminative pattern that can be used to classify patients. For this purpose, the process described in Villanueva *et al* [21] was modified as described below. The modified pipeline relies entirely on open source software and additional details are described in the bioinformatics section in File S1.

(1) A replicate summation and feature filter steps were added to consider zero values as special cases. Our original matrix contained a considerable amount of zero values for different features in different samples. Due to general limitation of MALDI technology, a significant fraction of these zero values could represent missing values rather than true zero intensities. To partially overcome this limitation we read each sample in replicates, and calculated the average intensity, ignoring zero intensity readings. Following this replicate summation, the resulted matrix still contained substantial amount of zero values. SVM-based models could classify according to zero values representing missing values and not true zero intensities. Therefore, we filtered out features that still had zero values in at least one of the samples. None of these removed features had clear preference of zero values to a specific clinical group assignment. The resulting sub-matrix was used in a machine learning classification.

(2) A new approach to feature selection parameterization was developed. The definitions for SVM-based analysis were initially as follows: RNTech stomach vs. RNTech control, Asterand stomach vs. Asterand control. Mann-Whitney p-value was calculated for each peak, according to clinical groups defined for the analysis. We then used Mann-Whitney p-values and peak intensities as cutoffs to select a subset of features (peaks) for usage in machine learning experiments. An intensity cutoff did not filter out samples in which at least one average reading had intensity above the cutoff for the peak tested. Filter values were optimized for best performance in SVM-based classifiers (produced by LIBSVM, linear kernel) according to ten-fold cross validation by a two-step protocol. The first step defined search ranges and intervals for both filters and iterating over all combinations. Then, the second step selected the combination of values, which provided the best performance and smallest number of features.

(3) A normalization step was added to control for cross-sample and cross-experiment biases. For sera sources' comparison and selection of features showing similar trends in both sources, cross-source normalization of intensities was performed using the R function “quantile” to define 9 thresholds *X_1_*_.*9*_ that divide the scaled values in the control class into 10 quantiles.

Additional bioinformatics methods are provided in File S1.

### Immuno-based commercial and clinical assays for the different apolipoproteins

ApoC-III and apoB-100 levels were measured by Immunoturbidometry on an Olympus 400 autoanalyzer, using the K-assay kits (cat # KAI-006 and 6142, Kamiya Biomedical, Seattle, WA, USA) as previously described [22]. In house ELISA for apoC-III is described in File S1. ApoC-I levels were tested using an AssayMax Human Apolipoprotein C-I ELISA kit (Assaypro, St. Charles, MO, USA) according to the manufacturer's instructions. Purified human apoC-I standards were included in the kit.

## Results

### Use of MS-based method to identify serum peptides signature for stomach cancer

Previous studies showed that well-designed and carefully-controlled sera peptidomics can separate specific cancer-bearing patients and non-cancer controls based on distinctive patterns of signature peptides in the serum [10], [11]. We investigated whether these results can be reproduced for stomach cancer and whether such separation is sufficient for analysis of sera from different sources. We first analyzed the serum peptide profiles of 62 patients with stomach cancer at different stages, as well as 41 control sera from healthy volunteers. These sera were obtained from two sources: (i) RNTech, a company that collected sera at Bucharest, Romania; and (ii) Asterand, a company that collected sera in the USA. For each source, the sera were collected using a single standard clinical protocol. The protocols were comparable e.g. the type of the tube, the clotting time and the initial freezing of the sera (see Methods), yet the blood withdrawal tubes were different. Age distribution, gender, and clinical characteristics of the 103 individuals included in this study are provided in Table 1 and in more detail in File S1. A summary of clinical stages of stomach cancer-derived sera for both sources is given in Table 1. Sample handling after the initial collection was uniform, involving 2 freeze-thaw cycles to accomplish initial storage and subsequent aliquoting for peptide extraction and MS analysis. All 103 serum samples were processed manually but identically employing one-step reverse-phase extraction. Sera samples and sample replicates were processed and read randomly on different dates to avoid preparation date-associated bias. All sera preparation and deposition was performed by the same individual. Similarly, all MALDI readings were performed by the same technician. The MALDI-TOF instrument's sensitivity was monitored routinely and constantly calibrated during all readings.

**Table 1:** Summary of clinical stages for stomach cancer and cancer-free individuals' sera from both sources.

	Total samples (MALDI study, Clinical study)				
	Number of samples				
Stage	Asterand	RNTech	Age average	Female	Male
**<IB**	8 (8,8)	0 (0,0)	57	6 (6,6)	2 (2,2)
**IB**	3 (3,3)	7 (7,7)	68	8 (8,8)	2 (2,2)
**II**	4 (4,4)	25 (16,22)	66	14 (11,14)	15 (9,12)
**IIA**	0 (0,0)	11 (0,11)	65	1 (0,1)	10 (0,9)
**IIIA**	2 (2,1)	16 (8,15)	67	5 (2,4)	13 (8,12)
**IIIB**	0 (0,0)	7 (3,4)	66	2 (2,1)	5 (1,3)
**IV**	5 (5,4)	13 (6,11)	60	7 (5,5)	11 (6,10)
**Stomach cancer**	22 (22,20)	79 (40,70)	64	43 (34,39)	58 (28,50)
**Controls**	21 (21,19)	33 (20,33)*	54	28 (22,26)	26 (19,25)

Asterand and RNTech clinical stages, age average and gender for stomach cancer and cancer-free individuals. The right and left values in parenthesis indicate the number of samples used for MALDI and clinical studies, respectively).

*RNTech control samples include 23 normal controls and 10 gastritis patients. Clinical information for each individual is depicted in File S1. The supplemental Tables include: sample ID, age at excision, sex, clinical diagnosis (specimen and patient), AJCC/UICC TNM classification, AJCC/UICC stage group, CRP values and CA 19-9 levels.

### Analysis of MS-based sera peptidome revealed a 9-peptide signature that distinguish stomach adenocarcinoma patients from cancer free controls

Total of 637 mass peaks (features) were identified in the 103 studied samples. The results of the MALDI were converted to a matrix containing the signal intensities of 637 mass peaks (features) for each of the studied serum samples with replicates for each sample (see methods, bioinformatics). While unsupervised hierarchical clustering using all features did not segregate cancer and non-cancer samples, PCA analysis of all features for each sera source differentiated between cancer and non-cancer samples (Figures S1–S3). This suggested that feature filtration and selection is essential before employing machine learning-based classification. Therefore we (i) applied a feature filtration and selection step and (ii) employed Mann-Whitney p-values and peak intensities as cutoffs to select a subset of features (peaks) for usage in machine learning experiments. (see methods, bioinformatics). We then analyzed within each source (RNTech and Asterand) whether sera of patients and controls could be segregated. We received good results for each of the single-source classifiers; SVM-based classifiers for RNTech and Asterand had 90.0% and 93.0% predicted accuracies, respectively, according to ten-fold cross validation of the training set (Table 2A). Random shuffling of group members resulted in much higher p-values (e.g. 0.8) and low predicted accuracy in trained models per each sera source. This indicated the significance of clinical conditions for classification into two clinically-defined groups within each sera source. However, the single-source classifiers did not perform well on the other source's samples, predicting correctly clinical status only in 35/60 samples (Asterand on RNTech) and 25/43 (RNTech on Asterand) (Table 2A). Therefore, source bias of peptidome has a significant effect on the accuracy of prediction.

**Table 2:** SVM-based models for single and mixed sera sources with and without cross-source normalization.

Normalization	Training set	Mann-Whitney p-value cutoff	Intensity filter cutoff	Number of features	Ten-fold cross validation predicted accuracy	Training controls identified by model	Training stomach cancer patients identified by model	Validation – second source controls identified	Validation- second source stomach cancer patients identified	Validation – third source controls identified	MCC for Training	MCC for Validation
**A.** No	Asterand	0.060	500	6	93.0%	21/21	22/22	13/20	22/40	1/12	1	−0.01
	RNTech	0.002	100	11	90.0%	20/20	40/40	3/21	22/22	12/12	1	0.5
	Mixed	0.044	100	9	84.1%	37/41	37/41	NA	13/21 #	10/12	0.81	0.44
**B.** Yes	Asterand	0.027	NA	9	93.0%	21/21	22/22	15/20	10/40	8/12	1	−0.04
	RNTech	0.001	NA	9	90.0%	20/20	40/40	17/21	14/22	11/12	1	0.50
	Mixed	0.008	NA	9	89.0%	38/41	36/41	NA	18/21 #	11/12	0.81	0.75

Normalization was performed according to control quantiles before averaging and feature selection. NA, not available.

#, 21 stomach cancer sera from RNTech and Asterand that are not included in the stomach cancer mixed sera set chosen by the SVM-based model for training.

**Figure 1:**
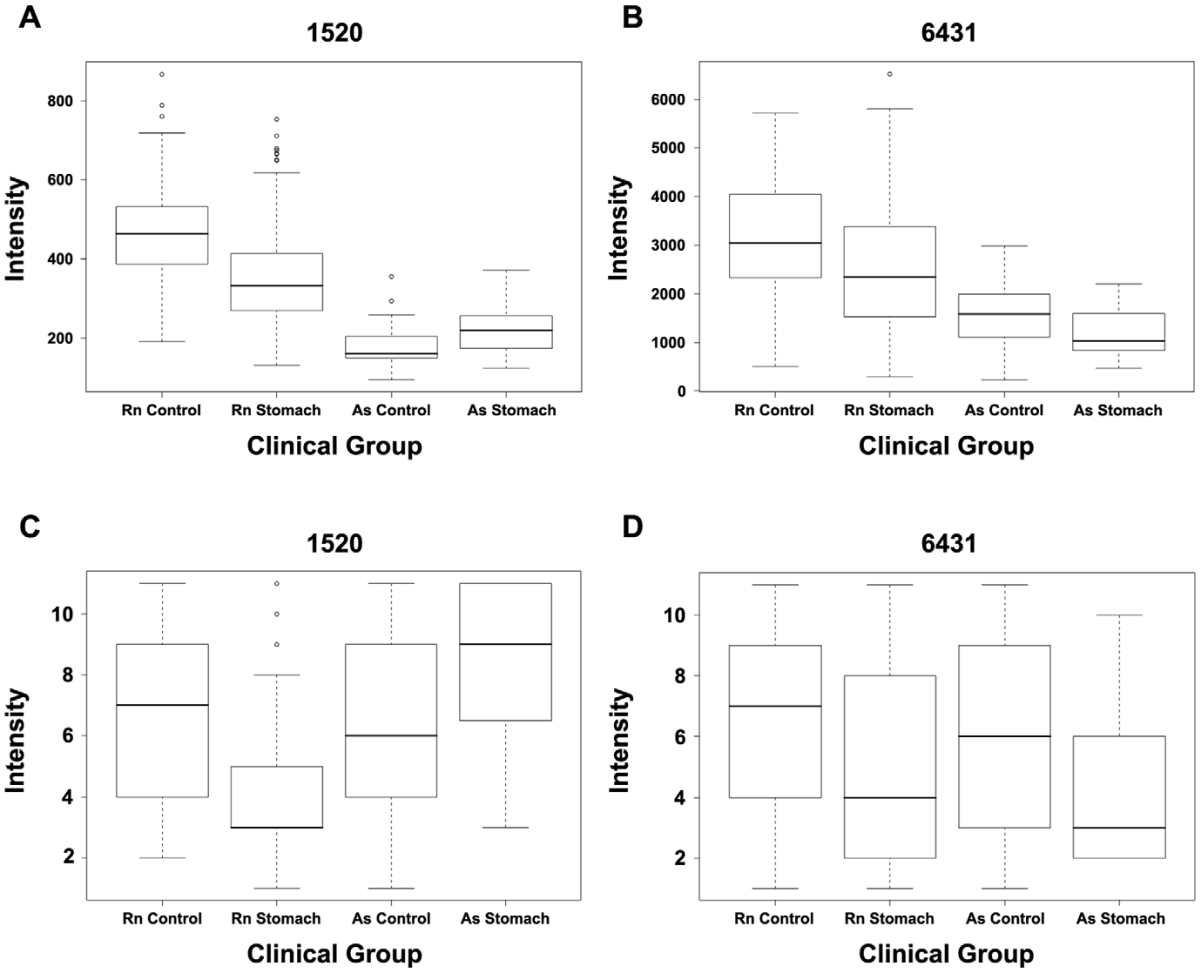
Boxplot presentations of features 1520 (A, C) and 6431 (B, D). A and B represent non-normalized peak intensities; C and D represent intensities following quantile normalization according to controls of each sera source (see methods). Rn, RNTech; As, Asterand.

The inability of models trained on one source to adequately predict clinical conditions from readings from the other source (Table 2A) is better presented when checking the features selected by the source-specific classifiers (Table 3). Some of the features which worked well on one source showed an opposite trend on the other source. Others were important for classification in one source but had little or no effect in the other. These observations led us to a comparative analysis of data from both sources. We produced box plots for all peak intensities, according to clinical groups. These plots showed that when comparing control and cancer intensities for each feature within a source, the trend observed could differ between the two sources (e.g. m/z 1520, Figure 1A). Even when the trend was persistent in both sources, the intensity values could be different (e.g. m/z 6431; RNTech higher than Asterand, Figure 1B). In order to create a prediction model, we needed to (i) discard source-specific phenomena, and (ii) add a normalization step which would reduce the effect of different intensity levels where the trend was maintained.

**Table 3:** Peaks selected for models when quantile normalization was used before averaging and feature selection.

m/z	Selected For	Bias Dist Rn	Bias Dist As	Bias Dist Mix	Possible identification
906	AM		12.44	4.017	V: m/z 905.5, FPA fragment FLAEGGGVR
1264	RM	77.57		1.935	V: m/z 1263.6, FPA fragment GEGDFLAEGGGVR
1351	R	−60.86			V: m/z 1350.6, FPA fragment SGEGDFLAEGGGVR
1520	AR	16.73	−6.35		V: m/z 1519.7, ITIH4 fragment GPPDVPDHAAYHPF
1534	A		−12.59		V: m/z 1533.79, b-ion of c3f SKITHRIHWESASLL
1549	A		8.51		?
4052	R	1.88			?
4088	RM	−28.53		2.666	EPO-KB: score 11, average m/z 4076.9, amyloid beta a4 protein
4207	RM	−0.48		−7.916	?
5752	ARM	−29.46	−0.8	−2.767	?
5902	A		−4.68		EPO-KB: score 3, average m/z 5902, fibrinogen alpha-chain frag.
6431	AM		12.57	2.799	EPO-KB: score 2.5, average m/z 6433.26, apolipoprotein C-I
6629	M			0.289	EPO-KB: score 5, average m/z 6624-6640, apolipoprotein C-I
9110	RM	13.08		−6.08	EPO-KB: score 20, average m/z 9130, haptoglobin
9443	ARM	8.43	−3.87	8.904	EPO-KB: score 0, average m/z 9443, apolipoprotein C-III
13997	A		6.73		?

List of the classifier-selected peaks for Asterand-based classifier (A), RNTech-based classifier (R) and Mixed set-based classifier (M). Models studied data obtained when quantile normalization was used before averaging and feature selection. The influence that each peak has on the prediction of the linear kernel SVM-based model was evaluated by entering an zero values vector and comparing this result to that of a vector containing zeros in all but one peak which has the maximum possible value. Directions were corrected so that signs would mean the same in all three models (minus: cancer; plus: control). Possible identification: Similar m/z values from the study by Villanueva *et al.* [11] and EPO-KB (searchable knowledge base of biomarker to protein links) [34] are presented.

The use of the mixed dataset with a Mann-Whitney p-value cutoff for feature selection could discard source-specific phenomena. Peaks which showed different trends in different sources would not be significant in the mixed set for clinical group-based separation; feature 1520 manifesting opposite trend between sources, was selected by each single source classifier (Figure 1A, Table 3). Therefore, it contributed to the lack of successful performance of each single source classifier on the other source (Table 2A). As expected, this feature was not selected by any model based on the mixed set. We created a mixed data set while randomly removing 21 stomach cancer samples from the mixed training set, and used these 21 removed samples for validation. In addition, we used the 12 cancer-free control samples collected in our laboratory as an independent control validation set. The model was selected in keeping with a maximum predicted accuracy according to a ten-fold cross validation, as before. The best scoring model for the mixed set was using 9 features (Mann-Whitney p-value filter of 0.044) and had a predicted accuracy of 84.1% according to ten-fold cross validation of the training set. Importantly, it accurately predicted 10/12 Israeli controls. However, this classifier predicted inadequately (13 of 21) the 21 removed mixed stomach cancer samples used for validation.

Therefore, to reduce the effect of source-related differences in intensity levels, the filter's performance in feature selection was enhanced by introducing a quantile normalization step. This normalization was performed according to controls of each sera source independently of the other sources (see methods, bioinformatics). For features, such as m/z 6431 with a persistent trend in both sources, this step corrected the intensity bias (Figure 1D). Indeed, 6431 feature was not selected for the non-normalized mix-based classifier. However, it was selected for the normalized mix-based classifier (Table 3). Yet, for features such as m/z 1520 with opposite trends in both sources, this step could not change the trend, as expected (Figure 1C).

We tested the quantile normalization's effect by applying it before averaging and feature selection. To better assess the prediction accuracy we employed the Matthews Correlation Coefficient (MCC) measure. MCC is used in machine learning as a measure of the quality of binary (two class) classifications and returns a value between −1 and +1. A coefficient of +1 represents a perfect prediction, 0 an average random prediction and −1 an inverse prediction. MCC is generally regarded as a balanced measure which can be used even if the classes are of different sizes. We thus calculated the MCC for various classification experiments in order to show the effect that normalization had on classification. Results are shown in Table 2. Note that without normalization, MCC was relatively high for the training set, yet showed mediocre performance on the validation set (Table 2). The normalization step gave similar high MCC values for training and validation sets (Table 2). The normalization step to control cross-source bias did not annul the need for machine learning-based classifier to define a discriminative pattern; PCA of the two sources-mixed normalized data sets resulted again in poor separation between stomach cancer and control samples (Figure S4).

### Immuno-based validation for features representing apoC-I and apoC-III

**Figure 2:**
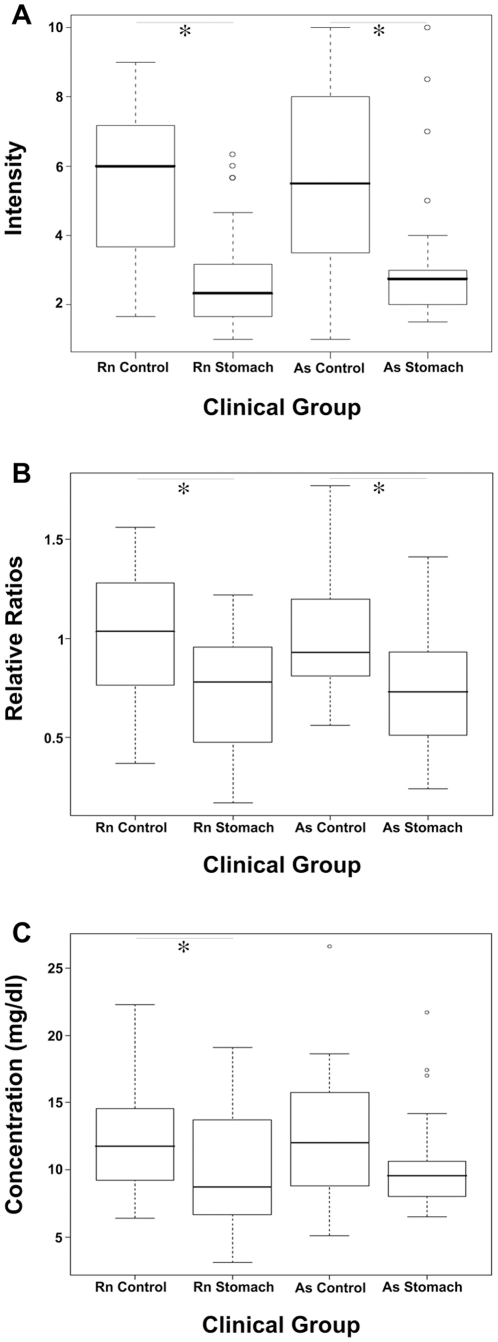
Boxplot presentations of MALDI feature 9443 and apoC-III ELISA assay. Boxplot presentations of MALDI feature 9443 (A), qualitative ELISA assay for apoC-III (B) and quantitative immunoturbidity-based assay for apoC-III (C). For A, units represent MALDI-based intensities following quantile normalization according to controls of each sera source (see methods). For B, units represent OD ratios of apoC-III ELISA following normalization to the average of controls of each sera source. For C, units represent apoC-III concentration in sera. For RNTech (Rn), * p-value <0.0001 for A and B, and <0.05 for C. For Asterand (As), * p-value <0.01 for A and B, and  = 0.06 for C; Wilcoxon rank sum test with continuity correction (alternative hypothesis: true location shift is greater than 0).

The classifier resulted from the mixed data set, following quantile normalization step, employed 9 features (Table 2). Three of the 9 features involved apolipoproteins: apoC-III (feature 9443) and apoC-I (features 6431 and 6629, Table 3). To further verify the MALDI-based results, we first developed an ELISA test for the qualitative detection of apoC-III in the serum (see methods) and tested all sera samples from Asterand and RNTech. Results of the ELISA followed the trend of the MALDI results (Figure 2A, B); Intensity of the apoC-III was significantly higher in control groups as compared to cancer groups in both sera sources. We further assayed the correlation between apoC-III ELISA and 9443 MALDI results per each sample; ELISA and MALDI results showed significant correlation (p<0.0001, Kendall's ran correlation tau). We then sent sera aliquots from nearly all samples (same freeze state) to an external clinical laboratory for immunoturbidity-based quantitative assay for apoC-III [22]. Results were obtained in mg/dl (Figure 2C) and as above, quantity of apoC-III was significantly higher in control groups of both sera sources.

**Figure 3:**
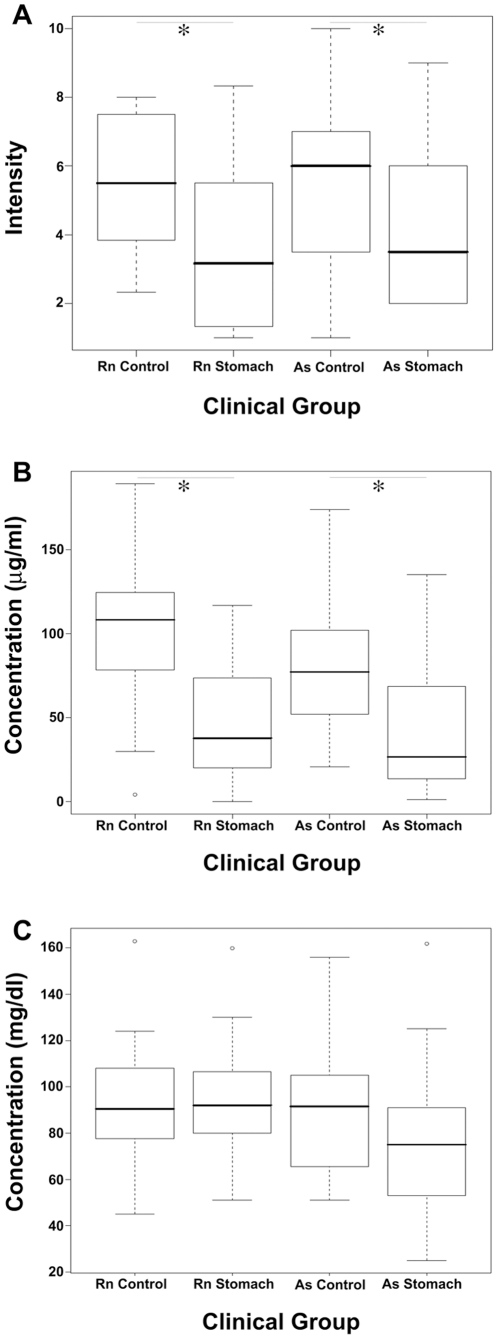
Boxplot presentations of MALDI feature 6629 and apoC-I and apoB-100 ELISA assay. Boxplot presentations of MALDI feature 6629 (A), quantitative ELISA assay for apoC-I (B) and quantitative immunoturbidity-based assay for apoB-100 (C). For A, units represent MALDI-based intensities following quantile normalization according to controls of each sera source (see methods). For B and C, units represent apoC-I and apoB-100 concentration in sera, respectively. For RNTech (Rn), * p-value  = 0.002 for A and <0.0001 for B. For Asterand (As), * p-value <0.05 for A and  = 0.001 for B; Wilcoxon rank sum test with continuity correction (alternative hypothesis: true location shift is greater than 0).

To verify the apoC-I MALDI results, we employed a commercial quantitative ELISA kit that includes apoC-I standards and recognizes both 6431 and 6629 variants of apoC-I. Results were obtained in µg/ml (Figure 3B) and followed the pattern observed for the MALDI results (Figures 1D and 3A); Intensity of the apoC-I was significantly higher in control groups as compared to cancer groups in both sera sources. To assess the specificity of apoC-I and apoC-III reduction in the sera of stomach cancer-bearing patients, we assayed apoB-100 levels. The samples assayed for apoC-III in the external clinical laboratory were assayed in parallel for apoB-100 levels using immunoturbidity-based quantitative assay. Results were obtained in mg/dl (Figure 3C) and showed no significant trend between control and stomach cancer-bearing groups. Therefore, we could make use of the apoB-100 results as a normalizing factor for the bioinformatics analysis of the quantitative apoC-I and apoC-III results (Figures 3C, 3B, 2C, respectively).

**Figure 4:**
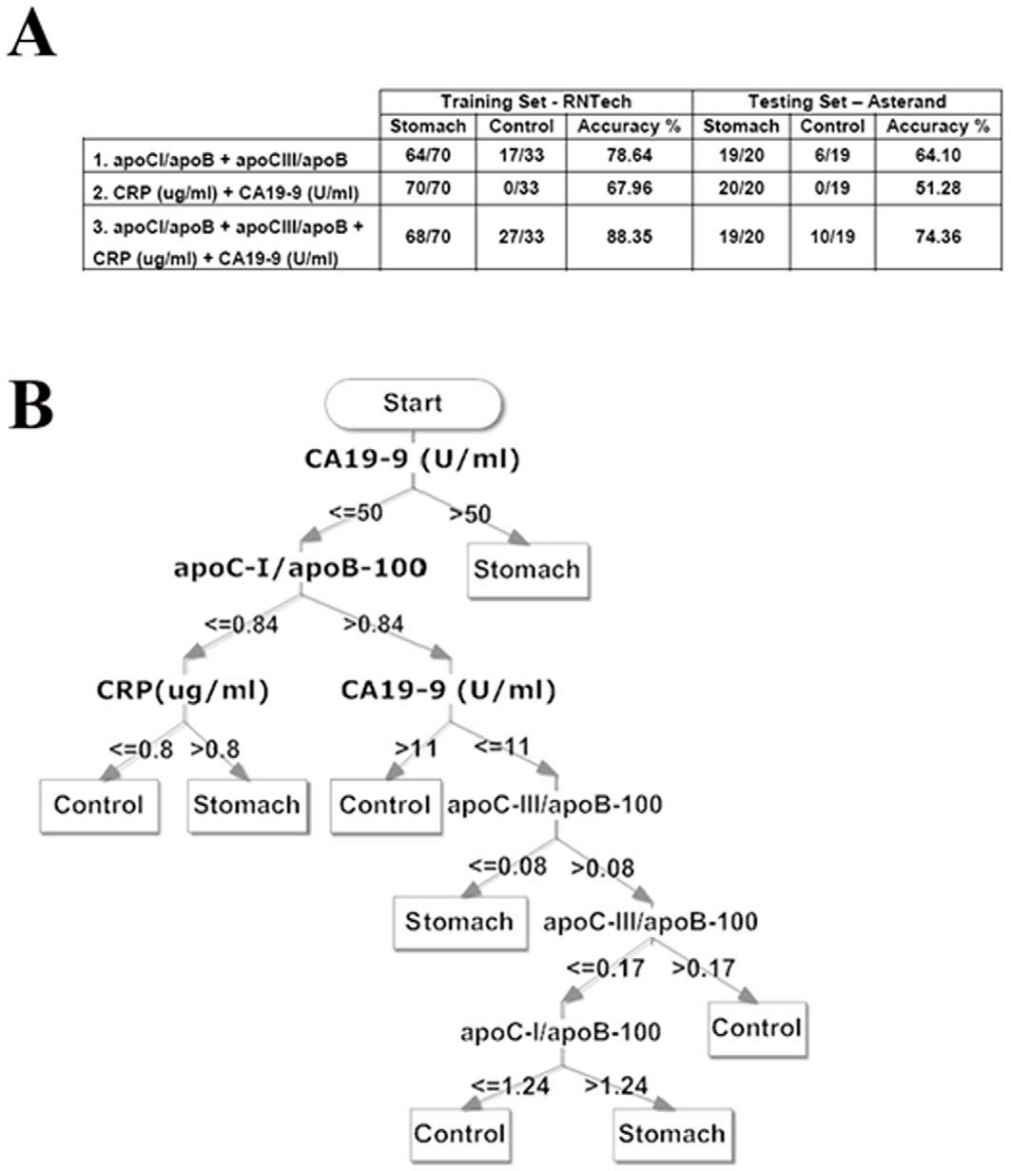
Decision tree for stomach cancer patients prediction and accuracy of prediction. (A) Accuracy of prediction produced by decision trees using (1) apoC-I/apoB-100 and apoC-III/apoB-100; (2) CRP (µg/ml) and CA19-9 (U/ml); and (3) apoC-I/apoB-100, apoC-III/apoB-100, CRP (µg/ml) and CA19-9 (U/ml) over training set RNTech and testing set Asterand. (B) Decision tree using the 4 features apoC-I/apoB-100, apoC-III/apoB-100, CRP (µg/ml) and CA19-9 (U/ml).

We analyzed clinically apoC-I, apoC-III and apoB-100 for additional samples from stomach cancer patients and cancer-free controls (RNTech source, same freeze-state; including 10 gastritis patients in the cancer-free controls; note Table 1 for the total sample numbers). We also analyzed clinically CA19-9 and CRP levels for all samples (same freeze-state). We then employed Clementine 10.0 software on the RNTech samples to assess whether rules set based on apoB-100-normalized C-I and C-III, CA19-9 and CRP serum levels can be used to classify between sera of control and stomach cancer groups of RNTech source as a training source. The combination of all 4 parameters yielded better prediction accuracy as compared to combination of less than 4 parameters (Figure 4 and data not shown). Prediction accuracy of the training set was 88.4%. We employed the RNTech-obtained rules set for the Asterand source and prediction accuracy was 74.4% (Figure 4). For both training and validation the sensitivity was excellent (87/90 combined) but the specificity was less accurate (37/52 combined).

## Discussion

In recent years, quite a few reports describing MS-identified serum biomarkers/signatures for cancerous states were proven wrong [5], [18]. Different sources of bias were described including sample selection, handling, processing, reading and analyzing [18], [20], [21]. Upon removal of bias-contributing factors, it was shown that SELDI-TOF MS whole serum proteomic profiling with IMAC surface did not reliably detect prostate cancer [23]. Therefore, the authors suggested that it is unlikely that a mass spectrometry approach using unprocessed serum would differentiate between men with and without prostate cancer [24]. On the other hand, other recent MALDI-TOF-based studies that avoided bias-contributing factors and employed a one-step sera processing technique identified discriminating biomarker signatures for different cancers including prostate cancer [11].

In this study we adopted the one step sera processing approach for identification of a peptidome-based signature to differentiate sera derived from stomach adenocarcinoma patients. We made a reasonable effort to avoid previously-reported bias contributing factors [18]. We analyzed sera from two biorepositories. We observed that even when sera handling, processing, MALDI reading and analysis are the same, peptidome analysis is biased by the biorepository. In addition to the socio-geographical differences (Romania and USA as the source for the samples in RNTech and Asterand, respectively), the source-related bias could be due to the brand of the blood withdrawal tube, used in the different biorepositories.

We then used a mixed sample set from two sera sources for feature selection and added a cross-source normalization step to compensate for source bias. We found that (i) the use of the mixed dataset with a Mann-Whitney p-value cutoff for feature selection could discard source-specific features, and (ii) a quantile normalization step helps to select (for machine learning) partially concordant features, in which the trends are concordant between sources, but intensity levels are different between sources. The need for normalization, when dealing with samples from different sources, was already shown for microarray-based high throughput technology [25]. It is well established that variations in experimental procedures and uncontrolled conditions (e.g. socio-geographical origin of samples) may lead to systemic measurement biases.

Following the modifications, we established a cross-source serum peptide signature for distinguishing stomach cancer patients from non-cancer controls. Three of the peptides corresponded to apoC-I and apoC-III. We validated our MALDI-based results with independent analytical methods that are based on immunoassays [26]. The peptide signature included apoC-III and apoC-I-derived features. The results from independent quantification of their serum levels followed the trend identified by the MS approach.

Our study is the first to report that serum levels of apoC-I and apoC-III can be used as potential biomarkers for stomach cancer. It is true that recent reports have indicated that apolipoproteins' levels in blood could be potential biomarkers for different cancers. ApoC-I was identified as a potential serum biomarker for colorectal cancer, hormone-refractory prostate cancer and liver fibrosis [27]–[29]. Other reports indicated that apoC-III might also be a potential biomarker in pancreatic cancer and breast cancer [30], [31]. However, all of these reports employed MALDI-based screening and did not verify their results with immuno-based or other assays. Nor did they study sera from another source as a validation group.

Our findings should be further expended and validated as described [32], [33]. Yet, the clinical validation of apoC-I and apoC-III results prompt us to further explore a diagnostic assay based on serum biomarkers that could be assayed in the clinic without the need for MS technology. Rules set utilizing apoB-100-normalized C-I and C-III, CA19-9 and CRP quantitative serum levels generated for the RNTech source and validated on the independent Asterand source had prediction accuracy of 88.4% and 74.4%, respectively. Therefore, the use of these 4 clinical features partially overcomes the source bias. However, the relatively lower specificity indicates that additional clinical parameter(s)/serum biomarkers should be added for the formulation of an applicable diagnostic score for stomach cancer patients. Additional source for such biomarkers could be differential glycosylation of secreted proteins that could provide additional serum biomarkers for cancerous state [35], [36].

## Supporting Information

File S1 Supplemental methods (bioinformatics, MALDI - serum preparation and reading). Supplemental results (hierarchical clustering and PCA). Legends to Figures S1 to S4 (showing hierarchical clustering and PCA). Tables S1 to S3, showing age distribution and gender, and clinical characteristics, are provided: Tables S1 (RNTech), S2 (Asterand) and S3 (Israeli controls).
(0.30 MB DOC)

Figure S1 Principal Components Analysis (PCA) on data derived from RNTech sera (A), Asterand sera (B), and the mixed (RNTech and Asterand) dataset (C). For A and B, blue and red circles indicate control and stomach samples, respectively. For C, green and red circles indicate Asterand control and stomach, respectively; black and blue indicate RNTech control and stomach, respectively.
(0.24 MB TIF)

Figure S2 Unsupervised hierarchical clustering on data derived from RNTech sera (A) and Asterand sera (B). For A, blue and black indicate cancer and control samples, respectively. For B, red and green indicate cancer and control samples, respectively.
(1.94 MB TIF)

Figure S3 Unsupervised hierarchical clustering on data derived from the mixed (RNTech and Asterand) dataset. For the RNTech samples set, blue and black indicate cancer and control samples, respectively. For the Asterand samples set, red and green indicate cancer and control samples, respectively.
(5.63 MB TIF)

Figure S4 Principal Components Analysis (PCA) on the normalized mixed (RNTech and Asterand) dataset. Green and red circles indicate Asterand control and stomach, respectively; black and blue indicate RNTech control and stomach, respectively.
(0.07 MB TIF)
