# Supplementary material for: Serum Apolipoproteins C-I and C-III Are Reduced in Stomach Cancer Patients: Results from MALDI-Based Peptidome and Immuno-Based Clinical Assays
Source: PLoS One. 2011 Jan 18;6(1):e14540. doi: 10.1371/journal.pone.0014540 (PMC3022591; doi:10.1371/journal.pone.0014540)
Supplement: File S1 — Supplemental methods (bioinformatics, MALDI - serum preparation and reading). Supplemental results (hierarchical clustering and PCA). Legends to Figures S1 to S4 (showing hierarchical clustering and PCA). Tables S1 to S3, showing age distribution and gender, and clinical characteristics, are provided: Tables S1 (RNTech), S2 (Asterand) and S3 (Israeli controls). (0.30 MB DOC) [file pone.0014540.s001.doc]

**SUPPLEMENTARY DATA**

**SUPPLEMENTAL METHODS**

**Serum sample processing for MS-MALDI**

Each serum sample was processed in two to three replicates (from identical aliquots and on separate random dates). 20 mg of beads coated with C8 (Ophir analytical, Rosh haAyn, Israel) per sample were mixed with 200 μl of pure Acetonitrile (ACN) (Sigma-Aldrich, St. Louis, MO, USA) and allowed to swell for 5 minutes, after which they were loaded onto a 96 well Ultra Screen plate (Millipore, Billerica, MA, USA) on a vacuum manifold (Millipore). A (-2) inch Hg vacuum was applied to clear the ACN from the wells. Two additional pre-washes with 200 μl of pure ACN were conducted, followed by two pre-wash steps with 0.1% (v/v) TFA (Mallinckrodt Baker B.V., Deventer, Netherlands) and another two of 200 μl of pure ACN. After 30 seconds of incubation with the pre-wash liquids, vacuum ((-2) to (-3) inch Hg) was applied to clear the wells during the pre-wash steps. A final pre-wash step with 50% ACN was applied. The resin was dried by a (-3) inch Hg vacuum for 1 minute. 50 μl aliquot of each serum sample was thawed on ice and centrifuged at 4^o^C, 13,000 g for 10 minutes. 30 μl from the mid-fraction was removed into a new lo-bind 1.5 ml tube (Eppendorf), mixed with 60 μl of freshly prepared 0.15% (v/v) TFA and immediately loaded onto the pre-washed C8 resin. The sample was incubated with the resin for 5 minutes and then a vacuum of (-3) inch Hg was applied to clear the wells. All samples were allowed to pass completely through the resin before proceeding to the wash steps. This was followed by 9 wash steps of 300 μl of 0.1% TFA, which was incubated for 30 seconds and then cleared using a (-3) inch Hg vacuum per wash. The resin was dried by a (-3) inch Hg vacuum for 10 minutes. The plate was located over a V-shape 96 well collecting plate (Greiner Bio One). The peptides were eluted with 50 μl of 80% ACN and a short spin (5810R, Eppendorf) of the plates (about 20 seconds) at room temperature. The eluted peptides were immediately transferred into a new lo-bind 1.5 ml tube and prepared for spotting onto a MALDI target plate.

**Sample preparation for MS-MALDI reading**

A saturated CHCA (puriss. p.a., matrix substance for MALDI-MS, CAS number [28166-41-8](http://www.sigmaaldrich.com/catalog/search/SearchResultsPage?Query=28166-41-8&Scope=CASSearch&btnSearch.x=1), Fluka, St. Louis, MO, USA) solution in 1:2 ACN:0.1% TFA was vortexed for 5 minutes and centrifuged for 1 minute at 10,000 g. The supernatant was transferred into a new lo-bind 1.5 ml tube. 5 μl of the matrix was mixed with 5 μl of the eluted peptides (see above), pipetted for 20 times and 1 μl spotted twice onto a stainless-steal MALDI target plate (Bruker Daltonics, Bremen, Germany). Each processed sample replicate was spotted in duplicate spots and read in a Reflex IV MALDI-TOF (Bruker Daltonics) on the same day.

**MS-MALDI-TOF reading**

Samples were analyzed on a Reflex IV MALDI-TOF instrument using a 337 nm radiation from a nitrogen laser VSL-337ND (Laser Science Inc, Franklin, MA, USA). The MALDI-TOF instrument's sensitivity was monitored routinely according to the manufacturer's instructions. Samples were spotted in a flower-like pattern, where the peptide standard (Bruker Daltonics) was spotted in the middle, surrounded by eight sample spots. The MALDI was calibrated according to the peptide standard, followed by the reading of the remaining eight spots. Each "flower" was calibrated again. Mass spectra, obtained from an average of up to 400 single-shot spectra of two target duplicate spots (2x100 single-shot from the first and 2x100 single-shot from the second spot) in reflectron mode (an accelerated voltage of 25 kV and a delay time of 200 ns) within a mass range from m/z 700 to 4100 Da. After this, the spectra were recorded in the same duplicate spots in a linear mode (an accelerated voltage of 20 kV, delay time of 200 ns) by averaging of 240 single shots (4x30 from the first spot and 4x30 single-shot from the second spot) within a mass range from m/z 2 kDa to 22 kDa.

**Bioinformatics**

For the generation of the intensity matrix from raw MALDI ASCII files, re-sampling was performed with SplineGUI.pl, a program written for this purpose, which performs Akima spline interpolation on the raw ASCII files. SplineGUI.pl was written in Perl [1], and uses the PDL (Perl Data Language [2]) and GSL (GNU Scientific Library [3]) libraries. Reflectron-generated ASCII files were interpolated from 900 to 4000 m/z and Linear-generated ASCII files were interpolated from 4000 to 20000 m/z. Alignment on the interpolated files was performed using Qcealignf, which performs spectra alignment employing Qpeaks for peak recognition [4]. This analysis produced two peak matrices (reflectron and linear) for all input files, which were merged into a single matrix containing all output peaks with 2-3 replicates for each serum sample.

An open source machine learning pipeline was developed, which we call m2pred.The entire analysis is preformed with m2pred, which was implemented in PERL, with calls to various accessory programs and R scripts ([http://www.r-project.org](http://www.r-project.org/)). The m2pred installation files, as well as the configuration file used to produce the results presented in this study are available upon request from Eitan Rubin at [erubin@bgu.ac.il](mailto:erubin@bgu.ac.il).

A replicate summation step was added, averaging non-zero values for a given m/z peak, except if all values were zero (in which case a value of zero was chosen). Zero values were further treated as special cases by removing peaks that had zero values (after replicate summation) in any sample.

For feature selection, the Villanueva *et al.* [4] approach was modified as follows: a battery of threshold values was constructed, involving different combination of a p value thresholds for the Mann-Whitney test and a minimal intensity threshold for intensity based filtering. The performance of thresholds combinations were compared by developing a machine learning model on a training set, using 10-fold cross validation to estimate the accuracy of the resulting model.

Feature selection and classifier development involved an improved approach to that is based on minimal intensity threshold and maximal Mann-Whitney p-value threshold. Rather than manually running the protocol with a single/limited possible sets of thresholds,[4] m2pred allowed us to search for the best combination of p-value and intensity cutoffs. This was performed by automatically studying a wide set of combinations generated by a user-defined range and intervals for each threshold. Then, for each combination, the entire machine learning process was executed. Classifier was developed using SVM with a linear kernel, as implemented in the LIBSVM library [5], called through the Algorithm::SVM Perl module. Data was scaled before using the SVM library as follows: intensity of each selected feature was scaled to a range of zero to hundred employing division by the maximal intensity value observed for relevant samples in the m/z matrix. The choice of best threshold combination for feature selection was primarily based on estimated classification accuracy, obtained with ten-fold cross-validation from the training set alone. However, a high priority was also given for a lower number of features used by the classifier to avoid over-fitting. As recommended,[4] selected features that were employed by the developed classifier were visually inspected to confirm results. Peak visualization was performed using peakView.pl, a Perl/Tk program we developed for this purpose, which draws the spectra in a defined range, color coded according to clinical information file, from Qcealignf output files.

**Bioinformatics: step of normalization for MS-MALDI analysis**

Manual inspection of the m/z profiles produced from sera originating from different sources revealed significant intensity biases between the sources. Therefore, to allow sera sources’ comparison and selection of features showing similar trends in both sources, cross-source normalization of intensities was performed using the following procedure (performed by m2pred): For each m/z value, the R function "quantile" was used to define 9 thresholds *X_1_*..*_9_* that divide the scaled values in the control class into 10 quantiles. Normalization of both control and cancer group (from the same sera source) intensities was thus achieved by replacing actual values *x* with their interval number *i*, using $\{(x,i)|X_{i-1}<x_{i}\leq X_{i}\}$ where$i=1..10$,$X_{0}=0$ and$X_{10}=1$. Zero values were kept to represent true zero intensity readings but were not used for quantile assessment. Box plots showing intensity values in each clinical group for all peaks, before and after normalization, were prepared using the R "boxplot" function. Normalization was performed before replicate summation and feature filtering and selection (see data analysis pipeline above). Therefore, for this type of normalization, feature selection involved only testing of a wide range of Mann-Whitney p-value threshold values, as threshold cutoff for normalized intensities became irrelevant. Alternatively, normalization was performed after feature selection and before the machine learning.

**Bioinformatics: additional data analysis tools**

In addition to the data analysis pipeline, m2pred also provides various data visualization tools including principal component analysis (PCA) and hierarchical clustering. PCA was performed using an R script employing the "prcomp" function with default parameters. The output is a 3D image file, in which each of the projection of each sample in the first three principal components is plotted, colored by clinical group. Hierarchical clustering was performed using an R script employing the "cor" function for calculating correlations (Pearson, Spearman and Kendall's tau) and the "hclust" function for clustering. Output to Newick format trees was performed by employing the "hc2Newick" function from the ctc package [6] available in BioConductor [7]. SVG format output was obtained using the "RSVGTipsDevice" R package. Alternatively, Newick files were opened using Dendroscope [8] and SVG files were outputted there. Node text colors were changed in SVG files using a Perl program.

**In house ELISA for apoC-III**

A 96 wells flat bottom MaxiSorp plate (NUNC, Rochester, NY, USA) was incubated over night with 300µl of 1% BSA (Sigma-Aldrich, St. Louis, MO, USA) in PBS (Gibco-BRL, Carlsbad, CA, USA) at 4^o^C. Using purified human apolipoprotein, we observed that coated BSA efficiently capture the apolipoproteins either purified or diluted into FCS. Thus we used coated BSA as a capture moiety for apolipoproteins in human serum. The BSA-coated plate was washed 3 times with PBS-T (0.05% Tween-20). Each serum sample was diluted 1:800 with dilution buffer (0.2% BSA in PBS-T) and 100μl were loaded onto the well for 1 hour incubation at 37^o^C. The plate was washed again for 3 times using PBS-T. A rabbit anti-human apoC-III (Meridian Life Science, Saco, ME, USA) was diluted 1:1,600 with dilution buffer. 100µl of the diluted antibody were added to the plate and incubated for 1 hour at 37^o^C. Three washes were preformed followed by 1 hour incubation at 37^o^C with 100µl of 1:1,000 diluted sheep rabbit HRP (GE Healthcare, Chalfont St. Giles, UK). The plate was washed 4 times and 100µl of room temperature (RT) TMB (DAKO, Glostrup, Denmark) were added and incubated at RT for 20 minutes and read using a Multiskan spectrum (Thermo electron, Waltham, MA, USA) ELISA plate reader at 650nm. This ELISA assay was specific for human apoC-III as signals obtained from bovine and mouse sera were similar to background signals from negative control (no sera) wells. The results from the chosen serum dilution were within the linear range of the ELISA, as determined by a titration curve.

**SUPPLEMENTAL RESULTS**

**PCA analysis differentiates stomach cancer and controls for each sera source but not for the mixed set**

The results of the MALDI were converted to a matrix containing the signal intensities of 637 mass peaks for each of the studied serum samples with 2-3 replicates per each sample. Principal component analysis (PCA) was used to compare the mass profiles of the samples (Figure S1). A good separation between stomach cancer and control samples was seen for each of the single-source data sets (Figure S1A, B). We received cumulative proportions of 87% and 84% for RNTech and Asterand, respectively that separated according to clinical condition (using 3 components PCA). However, PCA of the two sources-mixed data sets resulted in poor separation between stomach cancer and control samples. The cumulative proportion for 3 components was 89%. Yet, coloring the PCA derived from the mixed data set according to sera source (RNTech and Asterand) and clinical condition (cancer-bearing and control) showed that different sera sources, but not clinical condition, were separated in this analysis (Figure S1C). This indicated that most variances in the data set could be explained by sera source and not by other parameters such as clinical condition, gender or age.

**Hierarchical clustering did not differentiate stomach cancer and controls**

Following clinical group assignment and replicate summation, all 637 peaks for each of the 103 RNTech and Asterand samples were also taken for unsupervised hierarchical clustering. The resulting clusters showed a mixed pattern that did not differentiate disease from control or one sera source from the other (Figure S2). Similarly, when clustering analysis was performed for each sera source separately, no clear difference was observed between disease and control (Figure S2). Unsupervised hierarchical clustering also did not differentiate gender, age, time line of sample processing and reading. Recent studies have established distinctive serum polypeptide patterns through MS that reportedly correlate with clinically relevant outcomes (reviewed in [9]). In particular, Villanueva *et al* reported peptidome signature patterns for different solid tumor cancers [10, 11]. Unsupervised hierarchical clustering analysis of their data provided satisfactory aggregation without any prior statistical test [10, 11]. In contrast, unsupervised hierarchical clustering of our data resulted in a mixed pattern that did not differentiate disease from control (Figure S2).

The differences in unsupervised hierarchical clustering between our results for the stomach cancer study and the findings reported by Villanueva *et al* for other solid cancers [10, 11], could first be attributed to differences in virulence of the cancerous stages investigated. For example, we studied a stomach cancer group that consisted of only 11/62 (17.7%) patients defined as clinical stage IV (Table 1), while the study of metastatic thyroid cancer by Villanueva *et al* [10], had 30/60 (50%) of patients defined as stage IV. Alternatively, but not mutually exclusive, the quality of sample processing could attributed to the differences in the apparent segregation efficiencies. Application of peptidomics science and technologies to cancer biomarkers was limited due to poorly controlled variables in the clinical and analytical chemistry components and in MS signal processing [12]. Villanueva and colleagues evaluated, optimized and standardized a number of clinical and analytical chemistry variables that have been major sources of bias, consistency and variation among previous studies [4, 12]. We followed closely their suggested guidelines with regard to blood collection and clotting (e.g. tube, time and temperature), serum storage and handling (e.g. aliquots and two freeze-thaw cycle), crystallization and spectral acquisition [12]. We similarly employed Qpeaks and Qcealignf for signal processing and proper alignment as well as visualization tools [4]. Yet, the C8 beads employed for the peptide extraction method were different. Villanueva *et al.* employed one-step chromatography to desalt and extract low-molecular weight peptides, and clearly showed that the type, surface chemistries and batch of the reverse-phase chromatography resin are imperative for the quality and reproducibility of MS data [12]. We employed similar one-step reverse-phase chromatography but we could not obtain the batch of C8-magnetic beads employed by them. Consequently, we screened different types and batches of C8 beads (magnetic and non-magnetic), and similarly chose the C8 resin beads with the best performance. Therefore, though we tightly followed an optimized protocol for serum harvest, storage and processing, the chromatography resin beads employed by Villanueva and colleagues might have superior performance compared to our beads, and thus explain, in part, the differences in the results of hierarchical clustering. The lack of robotics in our study could also contribute to these differences.

**LEGENDS TO SUPPLEMENTAL FIGURES**

**Figure S1.** Principal Components Analysis (PCA) on data derived from RNTech sera (A), Asterand sera (B), and the mixed (RNTech and Asterand) dataset (C). For A and B, blue and red circles indicate control and stomach samples, respectively. For C, green and red circles indicate Asterand control and stomach, respectively; black and blue indicate RNTech control and stomach, respectively.

**Figure S2**. Unsupervised hierarchical clustering on data derived from RNTech sera (A) and Asterand sera (B). For A, blue and black indicate cancer and control samples, respectively. For B, red and green, indicate cancer and control samples, respectively.

**Figure S3**. Unsupervised hierarchical clustering on data derived from the mixed (RNTech and Asterand) dataset.
For the RNTech samples set, blue and black indicate cancer and control samples, respectively. For the Asterand samples set, red and green indicate cancer and control samples, respectively.

**Figure S4.** Principal Components Analysis (PCA) on the normalized mixed (RNTech and Asterand) dataset. Green and red circles indicate Asterand control and stomach, respectively; black and blue indicate RNTech control and stomach, respectively.

**Table S1.** Age distribution, gender and clinical characteristics of 60 sera samples from RNTech used for serum peptide profiling. All samples were collected and stored at Fundeni Clinical Institute Bucharest, Romania by RNTech company. Samples were shipped to Ben-Gurion University, Israel and processed following established protocols (see Methods). Abbreviations: AJCC/UICC TNM classification, American Joint Committee on Cancer (AJCC)/International Union against Cancer (UICC) tumor-node-metastasis (TNM) classification.

**Table S2.** Age distribution, gender and clinical characteristics of 43 sera samples from Asterand used for serum peptide profiling. All samples were collected and stored at the USA by Asterand company. Samples were shipped to Ben-Gurion University, Israel and processed following established protocols (see Methods). Abbreviations: AJCC/UICC TNM classification, American Joint Committee on Cancer (AJCC)/International Union against Cancer (UICC) tumor-node-metastasis (TNM) classification.

**Table S3.** Age distribution and gender of 12 sera samples from Israeli volunteers used for serum peptide profiling. All samples were collected, stored and processed at our lab following established protocols (see Methods).

**Table S1. Samples from RNTech**

| **Sample ID** | **Age at Excision** | **Sex** | **Clinical Diagnosis (specimen)** | **Clinical Diagnoses (patient)** | **AJCC/UICC TNM classification** | **AJCC/UICC Stage Group** | **CRP (µg/ml)** | **CA 19-9 (U/ml)** |
| --- | --- | --- | --- | --- | --- | --- | --- | --- |
| 30132 | 68 | Male | Adenocarcinoma of the stomach | severe anemia | T3N1M0 | IIIA | 70.4 | 2 |
| 30250 | 63 | Male | Adenocarcinoma of the stomach | _ | T3N2M0 | IIIB | 3.2 | 17 |
| 30317 | 69 | Male | Adenocarcinoma of the stomach | _ | T2bN1M0 | II | 62.5 | 67 |
| 30473 | 69 | Male | Adenocarcinoma of the stomach | hypertension, ischemic cardiophaty, leziune tricoronariana severa, prostate adenoma | T2bN1M0 | II | 89.3 | 4 |
| 30556 | 63 | Female | Adenocarcinoma of the stomach | cardiostimulator for sinusal node disease, mitral protesis for mitral disease, cronic atrial fibrialtion, IC cls II-III NYHA | T2AN1M0 | II | 2.7 | 15 |
| 30578 | 61 | Male | Adenocarcinoma of the stomach | _ | T2bN1M0 | II | 140.1 | 4 |
| 30620 | 65 | Female | Adenocarcinoma of the stomach | secondary anemia | T2aN1M1 | IV | 35.3 | 203 |
| 30623 | 62 | Male | Adenocarcinoma of the stomach | BPCO | T2aN2M0 | IIIA | 24.7 | 9 |
| 30634 | 59 | Male | Adenocarcinoma of the stomach | _ | T4N2M0 | IV | 15.9 | 171 |
| 30638 | 73 | Male | Adenocarcinoma of the stomach | hypertension | T2AN0M0 | IB | 1.7 | 3 |
| 40650 | 60 | Male | Adenocarcinoma of the stomach | _ | T2aN2M0 | IIIA | 8.1 | 11 |
| 40657 | 61 | Male | Adenocarcinoma of the stomach | secondary anemia, CICD angor de effort | T2bN1M0 | II | 10 | 4 |
| 40684 | 72 | Male | Adenocarcinoma of the stomach | hypertension | T2bN2M0 | IIIA | 4.2 | 9 |
| 40685 | 66 | Male | Adenocarcinoma of the stomach | easy anemia, cronic pancreatitis | T2bN2M0 | IIIA | 1.1 | 2 |
| 40706 | 68 | Female | Adenocarcinoma of the stomach | clecistectomy, liver steatosis | T2bN1M0 | II | 14.9 | 8 |
| 40732 | 63 | Male | Adenocarcinoma of the stomach | _ | T2bN1M0 | II | 7.7 | 9 |
| 40788 | 75 | Female | Adenocarcinoma of the stomach | hypochromic anemia, bilateral thoracal pachypleuritis, aortic atheromatosis, asthma | T2bN2M0 | IIIA | 22.9 | 5 |
| 40812 | 63 | Male | Adenocarcinoma of the stomach | secondary anemia, stroke, paintfull ischemic cardiophaty cls III NYHA, left bundle branch block | T2bN0M0 | IB | 8.6 | 3 |
| 40861 | 55 | Male | Adenocarcinoma of the stomach | hypertension, operated disk hernia, cardiopatie ischemica cronica,infectie cu VHB | T2aN0M0 | IB | 7.7 | 26 |
| 40884 | 64 | Female | Adenocarcinoma of the stomach | colecistectomy (2001) | T2bN2M1 | IV | 4.4 | 10 |
| 40906 | 70 | Female | Adenocarcinoma of the stomach | intestinal oclusion(1978), operated uterine fibroma(1980), hypertension | T2aN1M0 | II | 6.8 | 20 |
| 40918 | 58 | Male | Adenocarcinoma of the stomach | _ | T4N2M0 | IV | 55.8 | 55 |
| 40922 | 70 | Female | Adenocarcinoma of the stomach | mitral protesis, treated tuberculosis(at 25 years) | T3N2M0 | IIIB | 2.7 | 1 |
| 40937 | 67 | Male | Adenocarcinoma of the stomach | hypertension, left hydronephrosis degree I | T2aN0M0 | IB | 3.2 | 3 |
| 40953 | 65 | Male | Adenocarcinoma of the stomach | renal litiasis, gastric ulcer positive for Helycobacter pylori since 2002, secondary anemia | T3N3M0 | IV | 303.1 | 167 |
| 40959 | 65 | Male | Adenocarcinoma of the stomach | mitral failure, cardiac failure | T2bN0M0 | IB | 21.8 | 1 |
| 40972 | 74 | Male | Adenocarcinoma of the stomach | ischemic cardiophaty, hypertension stade III | T2bN2M0 | IIIA | 3.8 | 29 |
| 40996 | 75 | Female | Adenocarcinoma of the stomach | hypertension, ischemic cardiophaty, atherosclerosis, stroke | T2bN1M0 | II | 1.5 | 1 |
| 41011 | 63 | Male | Adenocarcinoma of the stomach | _ | T2bN1M0 | II | 2.4 | 9 |
| 41017 | 66 | Female | Adenocarcinoma of the stomach | ischemic cardiophaty, hypertension, operated uterine fibroma, hepatic cyst segment VII | T2aN1M0 | II | 5.2 | 31 |
| 41022 | 66 | Female | Adenocarcinoma of the stomach | duodenum ulcer, hypertension | T2bN1M0 | II | 5.6 | 7 |
| 41024 | 59 | Male | Adenocarcinoma of the stomach | disck hernia | T2aN2M0 | IIIA | 3.3 | 8 |
| 41033 | 74 | Male | Adenocarcinoma of the stomach | hypertension, CICND, IC cls II NYHA, insuficienta mitrala grad II apendicectomy, severe anemia | T2bN0M0 | IB | 31.4 | 3 |
| 41041 | 71 | Male | Adenocarcinoma of the stomach | gastric ulcer, anemia, hypertension, ischemic cardiophaty, leziuni bicoronariene la limita | T2bN1M0 | II | 2.7 | 28 |
| 51058 | 73 | Male | Adenocarcinoma of the stomach | _ | T2bN1M0 | II | 75.1 | 23 |
| 51085 | 77 | Female | Adenocarcinoma of the stomach | Apendicectomy; hypertension | T2aN1M0 | II | 0.4 | 8 |
| 51185 | 66 | Male | Adenocarcinoma of the stomach | operated right inghinal hernia, apendicectomy, secondary anemia | T2bN1M1 | IV | - | - |
| 51359 | 66 | Female | Adenocarcinoma of the stomach | extrasystolic arrhythmia, severe secondary anemia | T4N1M0 | IIIB | - | - |
| 51751 | 75 | Male | Adenocarcinoma of the stomach | toxic chronic hepatopatia | T2aN0M0 | IB | 7.0 | 2 |
| 51883 | 62 | Male | Adenocarcinoma of the stomach | - | T2N1M0 | II | - | - |
| 30227 | 66 | Male | Adenocarcinoma of the stomach | ulcerated blepharitis | T3N2M0 | IIIB | - | - |
| 30230 | 52 | Male | Adenocarcinoma of the stomach | _ | T3N0M0 | II | 39.9 | 17 |
| 30472 | 64 | Male | Adenocarcinoma of the stomach | _ | T2bN1M0 | II | - | - |
| 30625 | 45 | Male | Adenocarcinoma of the stomach | _ | T4N2M0 | IV | 38.5 | 203 |
| 40709 | 51 | Female | Adenocarcinoma of the stomach | hypertension, stroke | T4N0M0 | IIIA | 49.1 | 60 |
| 40767 | 52 | Male | Adenocarcinoma of the stomach | _ | T3N2M0 | IIIB | 63 | 253 |
| 40797 | 52 | Female | Adenocarcinoma of the stomach | iron deficency anemia | T2bN1M1 | IV | 4.3 | 1 |
| 40902 | 73 | Male | Adenocarcinoma of the stomach | cronic bronchopneumophaty (1954) | T3N1M0 | IIIA | 76 | 7 |
| 41009 | 63 | Male | Adenocarcinoma of the stomach | right fracture of lower limb | T2bN2M0 | IIIA | - | - |
| 41042 | 65 | Male | Adenocarcinoma of the stomach | operated perforated gastric ulcer (2000, adenocarcinoma) | T2bN0M0 | IIA | - | - |
| 41078 | 67 | Male | Adenocarcinoma of the stomach | _ | T2N1M0 | II | - | - |
| 51159 | 57 | Male | Adenocarcinoma of the stomach | erosive duodenitis, liver steatosis, piles, liver hemangioma segment VII | T3N2M0 | IIIB | - | - |
| 51219 | 73 | Male | Adenocarcinoma of the stomach | ischemic cardiophaty, apendicectomy, hypertension, extrasistolic aritmia, operated prostate adenoma (2000) | T2bN0M0 | IIA | 6.2 | 8 |
| 51226 | 46 | Male | Adenocarcinoma of the stomach | apendicectomy, unoperated gastric ulcer (2000),secondary anemia | T2bN2M0 | IIIA | 21.3 | 240 |
| 51406 | 70 | Male | Adenocarcinoma of the stomach | _ | T3N2M0 | IIIB | 34.8 | 4 |
| 51424 | 54 | Female | Adenocarcinoma of the stomach | uterine fibroma | T3N1M0 | IIIA | 5.3 | 9 |
| 51436 | 73 | Male | Adenocarcinoma of the stomach | colecistectomy, bilateral renal cysts | T2bN0M0 | IIA | 2.8 | 7 |
| 51440 | 73 | Male | Adenocarcinoma of the stomach | unpainful chronic ischemic heart disease, hypertension | T2bN1M0 | II | 40.9 | 148 |
| 51484 | 76 | Male | Adenocarcinoma of the stomach | hypertension | T2bN1M1 | IV | 234 | 158 |
| 51496 | 69 | Male | Adenocarcinoma of the stomach | glaucoma | T2bN0M0 | IIA | 9.3 | 1 |
| 51507 | 62 | Female | Adenocarcinoma of the stomach | hypertension | T2bN1M0 | II | 7.9 | 65 |
| 51558 | 54 | Male | Adenocarcinoma of the stomach | hypertension | T2bN2M0 | IIIA | 58 | 38 |
| 51577 | 67 | Male | Adenocarcinoma of the stomach | hypertension | T4N2M0 | IV | 0.7 | 3 |
| 51587 | 73 | Female | Adenocarcinoma of the stomach | secondary anemia | T2aN1M0 | II | 11.5 | 2 |
| 51644 | 65 | Male | Adenocarcinoma of the stomach | operated bilateral inguinal hernia | T2bN0M0 | IIA | 1.5 | 10 |
| 51660 | 56 | Female | Adenocarcinoma of the stomach | _ | T2bN3M0 | IV | - | - |
| 51685 | 69 | Female | Adenocarcinoma of the stomach | _ | T2aN1M0 | II | 3.5 | 4 |
| 51687 | 57 | Female | Adenocarcinoma of the stomach | secondary anemia | T2bN0M0 | IIA | 3.5 | 4 |
| 51700 | 59 | Male | Adenocarcinoma of the stomach | _ | T2aN1M0 | II | 3.4 | 6 |
| 51708 | 69 | Male | Adenocarcinoma of the stomach | treated lung tuberculosis, anemia | T2aN2M0 | IIIA | 4.6 | 252 |
| 51731 | 53 | Male | Adenocarcinoma of the stomach | painful ischemic chronic heart disease, hypertension, stroke (2004), pseudobulbar syndrom | T2bN0M0 | IIA | 0.5 | 2 |
| 51747 | 68 | Male | Adenocarcinoma of the stomach | painful ischemic chronic heart disease | T2aN0M0 | IIA | 19.3 | 6 |
| 51748 | 59 | Female | Adenocarcinoma of the stomach | secondary anemia | T3N1M0 | IIIA | 17.2 | 51 |
| 51759 | 73 | Male | Adenocarcinoma of the stomach | _ | T2bN0M0 | IIA | 3.6 | 4 |
| 51769 | 56 | Male | Adenocarcinoma of the stomach | hemangioma, gynecomastia | T2aN0M0 | IIA | 2.6 | 1 |
| 51797 | 59 | Male | Adenocarcinoma of the stomach | _ | T3N2M1 | IV | 10 | 204 |
| 51926 | 77 | Male | Adenocarcinoma of the stomach | hypertension, mitral disease | T2bN1M0 | II | 4.6 | 67 |
| 51973 | 71 | Male | Adenocarcinoma of the stomach | unpainful ischemic chronic heart disease, galucoma | T3N2M1 | IV | 115.5 | 10 |
| 51996 | 65 | Male | Adenocarcinoma of the stomach | colonic polyps | T2aN0M0 | IIA | 2.9 | 4 |
| 6015 | 65 | Female | Control donor | Diabetus mellitus, HTA, aortic stenosis |  |  | 0.4 | 9 |
| 6016 | 68 | Male | Control donor | - |  |  | 19.1 | 12 |
| 6017 | 75 | Male | Control donor | HTA |  |  | 3.1 | 1 |
| 6018 | 58 | Male | Control donor | - |  |  | 0.8 | 9 |
| 6019 | 56 | Male | Control donor | HTA |  |  | 14.6 | 8 |
| 6021 | 65 | Male | Control donor | HTA |  |  | 34.1 | 12 |
| 6022 | 66 | Female | Control donor | HTA |  |  | 1.9 | 7 |
| 6025 | 66 | Male | Control donor | Diabetus mellitus, HTA, inferior myocardial infarct sequela |  |  | 0.6 | 7 |
| 6026 | 66 | Male | Control donor | HTA |  |  | 4.2 | 16 |
| 6027 | 70 | Male | Control donor | Parkinson disease |  |  | 2 | 5 |
| 6028 | 69 | Female | Control donor | HTA, stroke |  |  | 4.8 | 28 |
| 6029 | 67 | Female | Control donor | HTA, stroke |  |  | 0.1 | 15 |
| 6030 | 76 | Male | Control donor | HTA, vertebro basilar stroke, sindrom pseudobulbar |  |  | 1 | 34 |
| 6031 | 67 | Female | Control donor | haemoragic stroke |  |  | 111.2 | 37 |
| 6032 | 71 | Female | Control donor | Diabetus mellitus, HTA, Parkinson disease, dislipidemia |  |  | 5.5 | 4 |
| 6034 | 70 | Female | Control donor | Diabetus mellitus, HTA, miltiple strokes |  |  | 6 | 3 |
| 6035 | 69 | Male | Control donor | cardiac failiure class IV NYHA, HTTP biologic mitral prosthesis |  |  | 15 | 4 |
| 6036 | 73 | Female | Control donor | HTA |  |  | 1.4 | 7 |
| 6037 | 65 | Male | Control donor | recent myocardial infarct, angor rezidual |  |  | 1.8 | 0 |
| 6041 | 72 | Female | Control donor | HTA |  |  | 0.3 | 17 |
| 6088 | 40 | Male | Control donor | - | - | - | 5.8 | 8 |
| 6090 | 43 | Female | Control donor | - | - | - | 9.2 | 0 |
| 6094 | 40 | Female | Control donor | - | - | - | 7.9 | 8 |
| 30298 | 74 | Male | Gastritis | duble active ulcer | - | - | 4.8 | 4 |
| 30453 | 73 | Female | Gastritis | stromal tumour with fusiform cells, hypertension, liver steatosis, bilateral renal microlithiasis | - | - | 3.7 | 50 |
| 30461 | 68 | Male | Gastritis | active ulcer | - | - | 2.5 | 1 |
| 40842 | 24 | Male | Gastritis | stromal tumour with fusiform cells, apendicetomy, hypertension, liver steatosis degree I, left renal microlithiasis | - | - | 8.6 | 11 |
| 40928 | 66 | Male | Gastritis | active ulcer, severe secondary anemia | - | - | 6.1 | 7 |
| 51238 | 59 | Male | Gastritis | inflamator fibroid polyp, hypertension, ischemic cardiophaty, stroke(1998), left inghinal hernia, anemia | - | - | 7.5 | 3 |
| 51374 | 64 | Male | Gastritis | stromal tumour with epitelioid cells, low malignity (GIST), painful chronic ischemic heart disease, supraventricular extrasistolic aritmia, bilareral renal microlithiasis | - | - | 0.5 | 8 |
| 51733 | 54 | Female | Gastritis | chronic gastric ulcer, secondary anemia | - | - | 6.9 | 14 |
| 62146 | 52 | Male | Gastritis | gastric schwannoma, renal litiasis | - | - |  |  |
| 62221 | 55 | Male | Gastritis | gastric stromal tumour with low malignity risk | - | - | 8.4 | 3 |

**Table S2. Samples from Asterand**

| **Sample ID** | **Age at Excision** | **Sex** | **Clinical Diagnosis (specimen)** | **Clinical Diagnoses (patient)** | **AJCC/UICC TNM classification** | **AJCC/UICC Stage Group** | **CRP (µg/ml)** | **CA 19-9 (U/ml)** |
| --- | --- | --- | --- | --- | --- | --- | --- | --- |
| 17615A2 | 60 | Male | Adenocarcinoma of the stomach | Adenocarcinoma of the stomach(Status:New) |  |  | 1.4 | 6 |
| 19872A10 | 60 | Male | Carcinoma of the stomach | Hypertension(Status:Ongoing), Diabetes(Status:Ongoing), Peptic ulcer disease(Status:Ongoing), Depression(Status:Ongoing), Hiatal hernia(Status:Past), Carcinoma of the stomach(Status:New), Diabetes: TypeII |  |  | 81.5 | 3 |
| 19956A2 | 65 | Female | Adenocarcinoma of the stomach | Coronary artery disease(Status:Ongoing), Carcinoma of stomach(Status:New) | TXNXMX |  | 79.5 | 3 |
| 19968A3 | 50 | Female | Adenocarcinoma of the stomach | Chronic gastritis(Status:Ongoing), Adenocarcinoma of the stomach(Status:Ongoing) | T3NXMX | II | 139.1 | 101 |
| 24159A5 | 84 | Male | Adenocarcinoma of the stomach | Colon cancer(Status:Past), Cardiac arrythmia(Status:Ongoing), Hypertension(Status:Ongoing), Adenocarcinoma of the stomach(Status:New) | T1N1MX | IB | 273.1 | 5 |
| 31517A3 | 47 | Female | Adenocarcinoma of the stomach | Obesity(Status:Ongoing), Adenocarcinoma of the stomach(Status:Ongoing) | TXN1M0 |  | 62.8 | 12 |
| 31519A3 | 57 | Female | Adenocarcinoma of the stomach | Chronic cholecystitis(Status:Ongoing), Adenocarcinoma of the stomach(Status:New) | T2N1M0 | II | 67.3 | 8 |
| 31522A3 | 74 | Female | Adenocarcinoma of the stomach | Coronary artery disease(Status:Ongoing), Adenocarcinoma of the stomach(Status:New) | TXN1M1 | IV | 100 | 40 |
| 31526A2 | 63 | Female | Adenocarcinoma of the stomach | Coronary artery disease(Status:Ongoing), Adenocarcinoma of the stomach(Status:New) | TXNXM0 |  | 3.8 | 139 |
| 31532A2 | 39 | Female | Adenocarcinoma of the stomach | Adenocarcinoma of the stomach(Status:New) | TXN1M0 |  | 38.7 | 11 |
| 31535A3 | 50 | Female | Adenocarcinoma of the stomach | Chronic gastritis(Status:Ongoing), Adenocarcinoma of the stomach(Status:New) | TXN1M0 |  | 93.3 | 7 |
| 31550A4 | 64 | Male | Non-differentiated carcinoma of stomach | Acute respiratory viral infection(Status:Past), Ischemic heart disease(Status:Ongoing), Non-differentiated carcinoma of stomach(Status:New) | T3N1M0 | IIIA | 156.3 | 63 |
| 31551A3 | 56 | Male | Adenocarcinoma of the stomach | Cholelythiasis(Status:Ongoing), Pneumonia(Status:Past), Chronic bronchitis(Status:Ongoing), Adenocarcinoma of the stomach(Status:New) | T3N1M1 | IV | 1.5 | 4 |
| 31559A2 | 73 | Female | Adenocarcinoma of the stomach | Arterial hypertension(Status:Ongoing), Chronic pyelonephritis(Status:Ongoing), Asymptomatic varicose veins(Status:Ongoing), Adenocarcinoma of the stomach(Status:New) | T3N0MX | II | 179.6 | 14 |
| 31564A2 | 57 | Male | Adenocarcinoma of the stomach | Acute respiratory virus disease(Status:Past), Adenocarcinoma of the stomach(Status:New) | TXN1M1 | IV | 390.3 | 8 |
| 31570A2 | 49 | Female | Adenocarcinoma of the stomach | Acute respiratory virus disease(Status:Ongoing), Adenocarcinoma of the stomach(Status:Ongoing) | T2N0M0 | IB | 341.6 | 13 |
| 31593A2 | 70 | Female | Adenocarcinoma of the stomach | Coronary artery disease(Status:Ongoing), Adenocarcinoma of the stomach(Status:New) | TXN1M0 |  | 1.3 | 9 |
| 31600A2 | 62 | Female | Adenocarcinoma of the stomach | Essential hypertension(Status:Ongoing), Adenocarcinoma of the stomach(Status:New) | T3NXM0 | II | 2.4 | 34 |
| 51931A1 | 49 | Female | Signet ring cell carcinoma of stomach metastatic to ovaries | Chronic gastritis(Status:Ongoing), Signet ring cell carcinoma of stomach metastatic to ovaries(Status:Ongoing) | TXNXM1 | IV | 1.4 | 6 |
| 74334A1 | 75 | Female | Adenocarcinoma of the stomach | Arthritis - hips(Status:Ongoing), Non-insulin dependent diabetes mellitus (Type II)(Status:Ongoing), Coronary artery disease(Status:Ongoing), Hypertension(Status:Ongoing), Adenocarcinoma of the stomach(Status:New), Diabetes: Type II | T2bN0MX | IB | 16.4 | 38 |
| 62922A3 | 70 | Female | Adenocarcinoma of the stomach | Adenocarcinoma of the stomach(Status:New), Myocardial infarction(Status:New), Arthritis(Status:Ongoing), Diabetes(Status:Ongoing), Hypertension(Status:Ongoing) | T3N1MX | IIIA | - | - |
| 57414A2 | 44 | Female | Adenocarcinoma of the stomach metastatic to ovary | Recovering alcoholic(Status:Past), Liver cancer with metastasis to the bone(Status:Ongoing), Adenocarcinoma of the stomach metastatic to ovary(Status:New) | TXNXM1 | IV | - | - |
| 60402A2 | 24 | Female | Control donor | Control donor(Status:New) | Control |  | 58.7 | 9 |
| 60407A2 | 50 | Female | Control donor | Control donor(Status:New) | Control |  | 7.8 | 13 |
| 60421A2 | 23 | Female | Control donor | Control donor(Status:New) | Control |  | - | - |
| 60429A4 | 29 | Female | Control donor | Mitral valve prolapse(Status:Ongoing), Control donor(Status:New) | Control |  | 0 | 8 |
| 60958A2 | 51 | Female | Control donor | Migraines(Status:Past), Control donor(Status:New) | Control |  | 0.9 | 7 |
| 60964A2 | 51 | Male | Control donor | Control donor(Status:New) | Control |  | 0.7 | 8 |
| 60975A10 | 30 | Female | Control donor | Control donor(Status:New) | Control |  | 5.7 | 13 |
| 61304A3 | 30 | Male | Control donor | Control donor(Status:New) | Control |  | 0.4 | 8 |
| 61310A10 | 47 | Female | Control donor | Control donor(Status:New) | Control |  | 0.4 | 10 |
| 61645A2 | 57 | Male | Control donor | Control donor(Status:Ongoing) | Control |  | 0.1 | 14 |
| 61683A2 | 49 | Female | Control donor | Control donor(Status:New) | Control |  | 4.5 | 10 |
| 61699A2 | 59 | Male | Control donor | Control donor(Status:New) | Control |  | 1.5 | 8 |
| 62615A6 | 25 | Female | Control donor | Control donor(Status:New) | Control |  | 5.2 | 6 |
| 63327A7 | 33 | Female | Normal control donor |  | Control |  | - | - |
| 63341A6 | 20 | Female | Control donor | Myringotomy(Status:Past), Control donor(Status:New) | Control |  | 0.7 | 2 |
| 63375A6 | 41 | Female | Normal control donor |  | Control |  | 0.1 | 1 |
| 63379A6 | 38 | Female | Normal control donor |  | Control |  | 0.2 | 2 |
| 85917A6 | 33 | Female | Control donor | Control donor(Status:New) | Control |  | 0.3 | 5 |
| 85941A6 | 55 | Male | Normal control donor |  | Control |  | 1.3 | 8 |
| 85949A6 | 63 | Male | Control donor | Control donor(Status:New) | Control |  | 3.8 | 2 |
| 85954A6 | 53 | Female | No current illness | Arthritis(Status:Ongoing), Hypercholesterolemia (Status:Ongoing), Sinus problems(Status:Ongoing), No current illness(Status:) | Control |  | 1.3 | 13 |

**Table S3. Israeli controls samples.**

| **Sample ID** | **Age at Blood Withdrawal** | **Sex** |
| --- | --- | --- |
| 16 | 25 | Female |
| 18 | 37 | Male |
| 20 | 31 | Male |
| 30 | 54 | Male |
| 32 | 36 | Male |
| 33 | 28 | Male |
| 34 | 61 | Female |
| 35 | 50 | Female |
| 36 | 55 | Female |
| 37 | 24 | Female |
| 39 | 32 | Male |
| 40 | 39 | Male |

References:

1 Wall L, Christiansen T, Orwant J. Programming PERL.

2 Glazebrook K, Economou F. The PERL journal 1997;**5**.

3 Galassi M, Davies J, Theiler J, Gough B, Jungman G, Booth M*, et al.* GNU scientific library reference manual.

4 Villanueva J, Philip J, DeNoyer L, Tempst P. Data analysis of assorted serum peptidome profiles. Nat Protoc 2007;**2**:588-602.

5 M'Koma A E, Blum DL, Norris JL, Koyama T, Billheimer D, Motley S*, et al.* Detection of pre-neoplastic and neoplastic prostate disease by MADI profiling of urine. Biochem Biophys Res Commun 2007;**353**:829-34.

6 Lucas A, Jasson S. Using amap ctc package for huge clustering. R News 2006;**6**:58-60.

7 Gentleman RC, Carey VJ, Bates DM, Bolstad B, Dettling M, Dudoit S*, et al.* Bioconductor: open software development for computational biology and bioinformatics. Genome Biol 2004;**5**:R80.

8 Huson DH, Richter DC, Rausch C, Dezulian T, Franz M, Rupp R. Dendroscope: An interactive viewer for large phylogenetic trees. BMC Bioinformatics 2007;**8**:460.

9 Petricoin EF, Belluco C, Araujo RP, Liotta LA. The blood peptidome: a higher dimension of information content for cancer biomarker discovery. Nat Rev Cancer 2006;**6**:961-7.

10 Villanueva J, Martorella AJ, Lawlor K, Philip J, Fleisher M, Robbins RJ*, et al.* Serum peptidome patterns that distinguish metastatic thyroid carcinoma from cancer-free controls are unbiased by gender and age. Mol Cell Proteomics 2006;**5**:1840-52.

11 Villanueva J, Shaffer DR, Philip J, Chaparro CA, Erdjument-Bromage H, Olshen AB*, et al.* Differential exoprotease activities confer tumor-specific serum peptidome patterns. J Clin Invest 2006;**116**:271-84.

12 Villanueva J, Philip J, Chaparro CA, Li Y, Toledo-Crow R, DeNoyer L*, et al.* Correcting common errors in identifying cancer-specific serum peptide signatures. J Proteome Res 2005;**4**:1060-72.
