# Supplementary figures and images for: Serum Apolipoproteins C-I and C-III Are Reduced in Stomach Cancer Patients: Results from MALDI-Based Peptidome and Immuno-Based Clinical Assays
Source: PLoS One. 2011 Jan 18;6(1):e14540. doi: 10.1371/journal.pone.0014540 (PMC3022591; doi:10.1371/journal.pone.0014540)

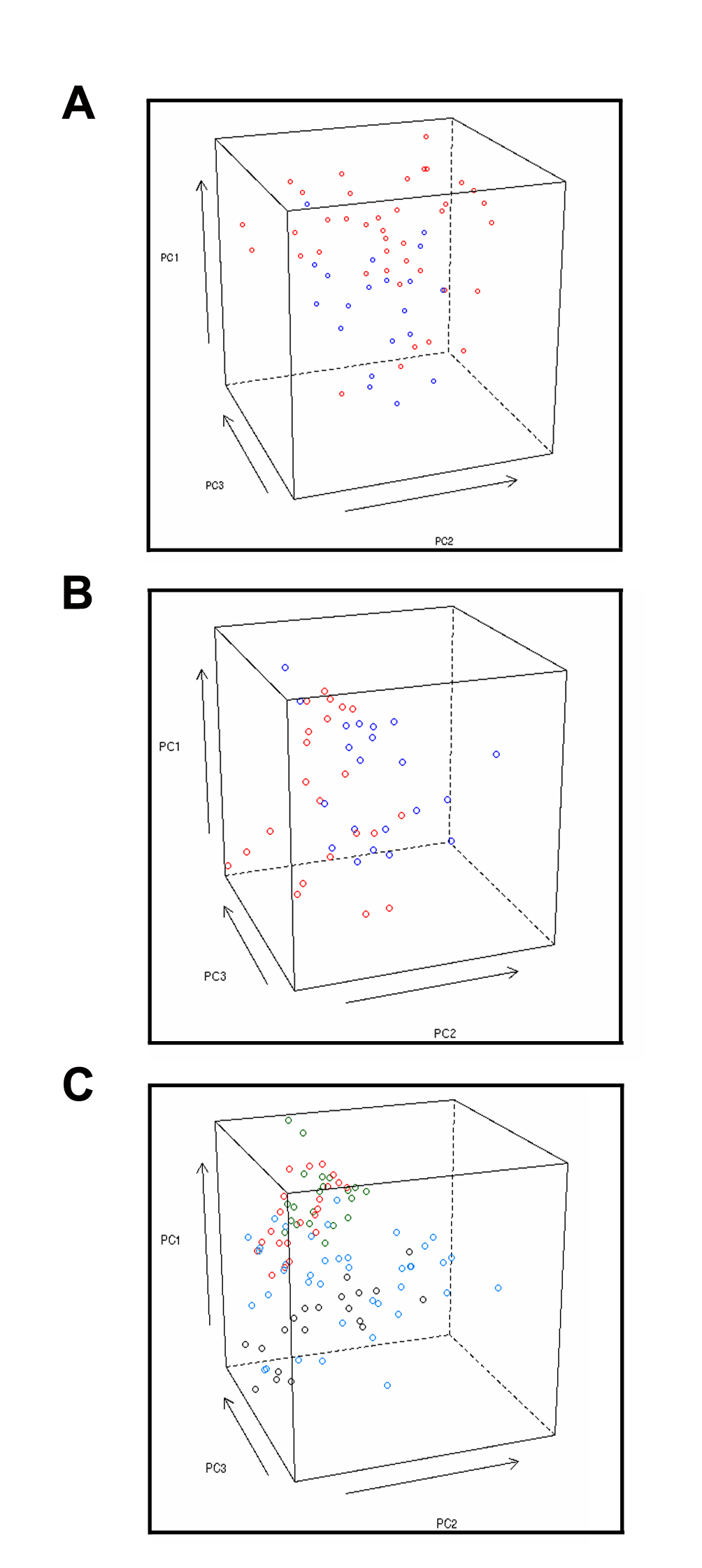

Supplement: Figure S1 — Principal Components Analysis (PCA) on data derived from RNTech sera (A), Asterand sera (B), and the mixed (RNTech and Asterand) dataset (C). For A and B, blue and red circles indicate control and stomach samples, respectively. For C, green and red circles indicate Asterand control and stomach, respectively; black and blue indicate RNTech control and stomach, respectively. (0.24 MB TIF) [file pone.0014540.s002.tif]

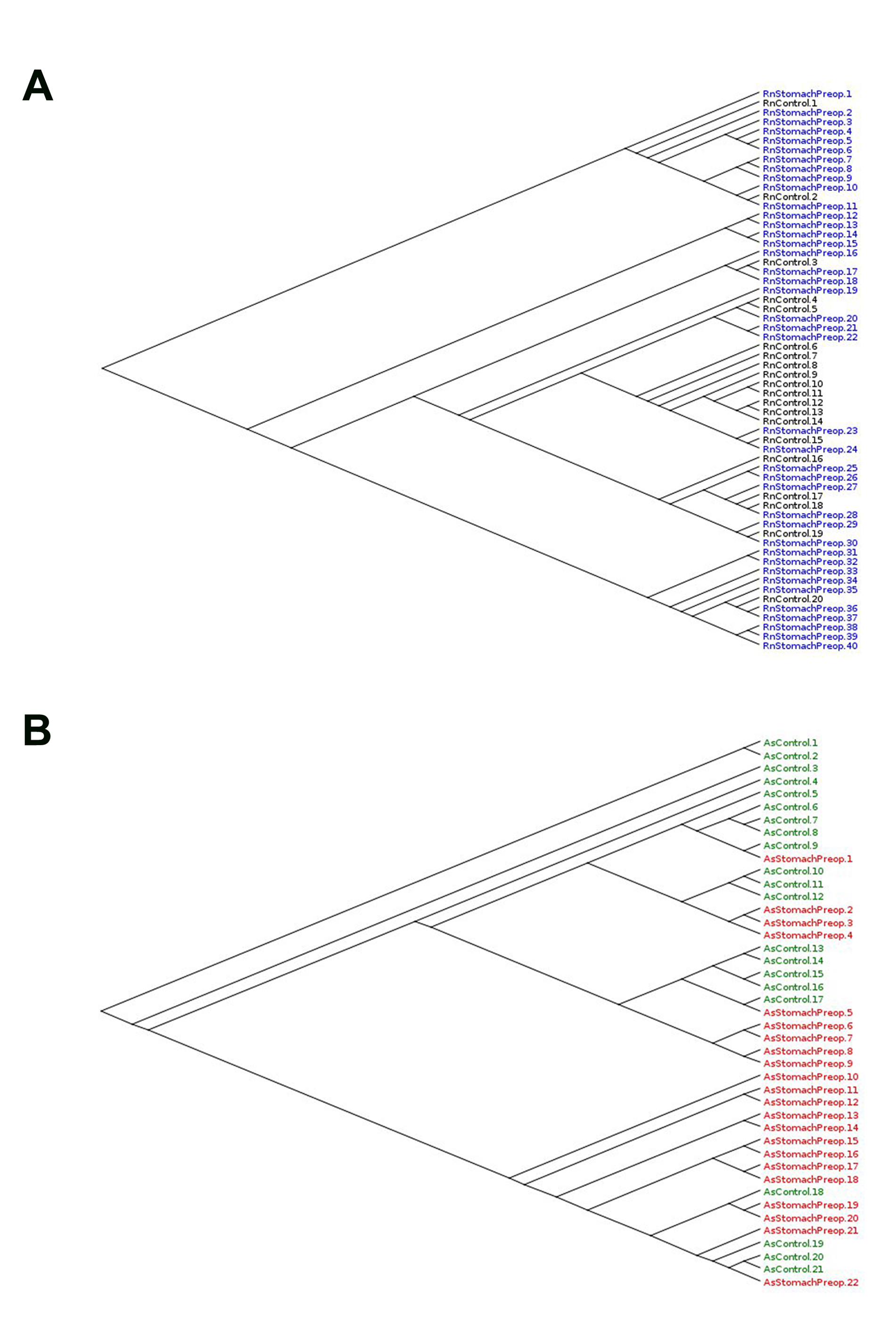

Supplement: Figure S2 — Unsupervised hierarchical clustering on data derived from RNTech sera (A) and Asterand sera (B). For A, blue and black indicate cancer and control samples, respectively. For B, red and green indicate cancer and control samples, respectively. (1.94 MB TIF) [file pone.0014540.s003.tif]

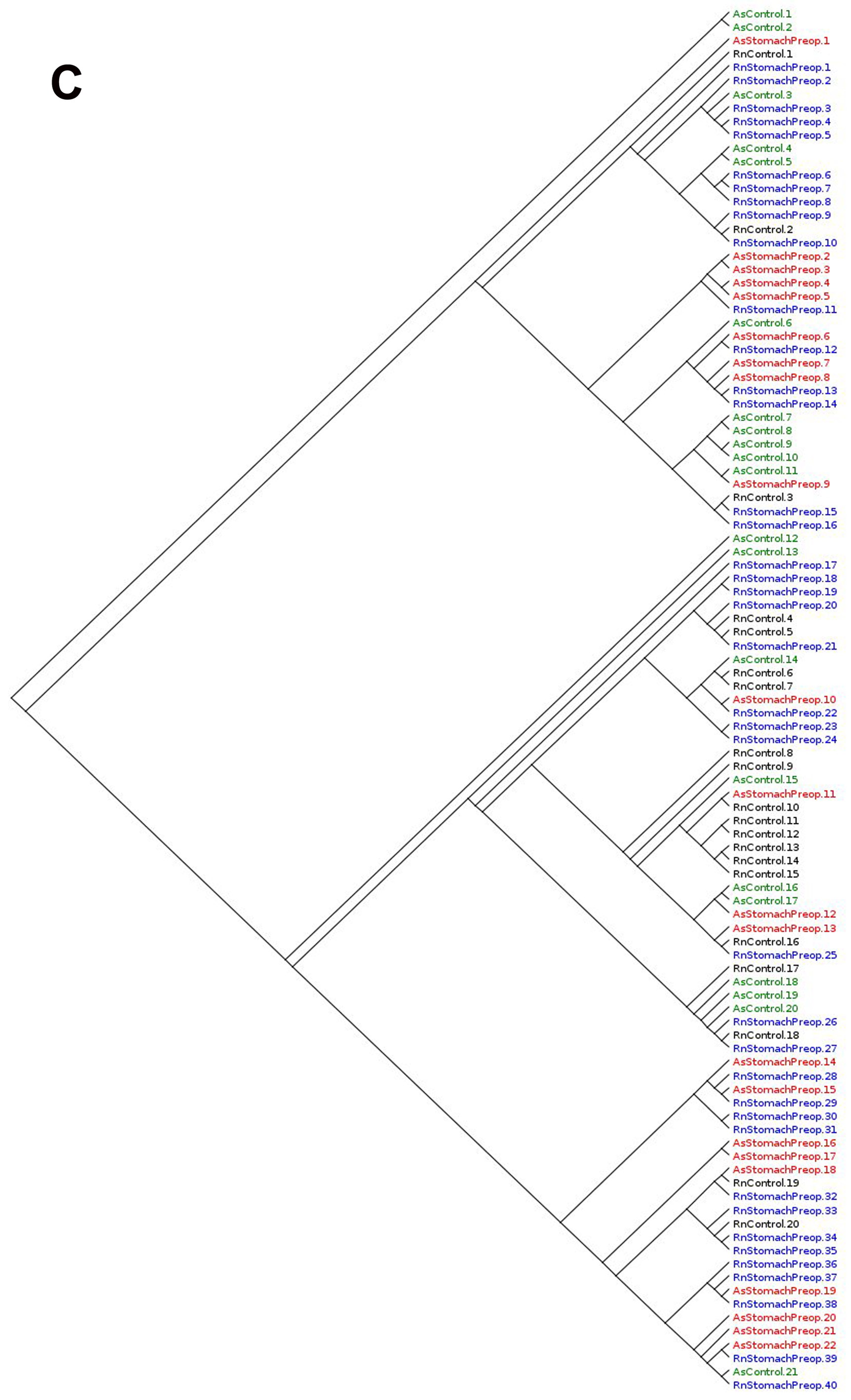

Supplement: Figure S3 — Unsupervised hierarchical clustering on data derived from the mixed (RNTech and Asterand) dataset. For the RNTech samples set, blue and black indicate cancer and control samples, respectively. For the Asterand samples set, red and green indicate cancer and control samples, respectively. (5.63 MB TIF) [file pone.0014540.s004.tif]

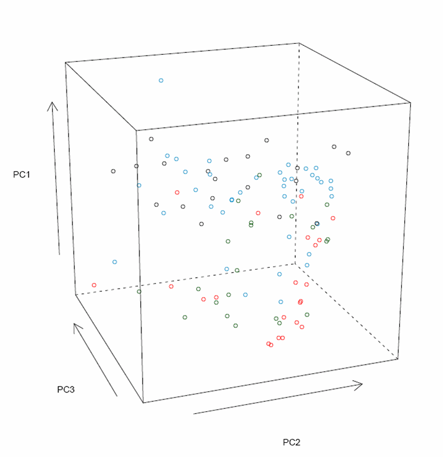

Supplement: Figure S4 — Principal Components Analysis (PCA) on the normalized mixed (RNTech and Asterand) dataset. Green and red circles indicate Asterand control and stomach, respectively; black and blue indicate RNTech control and stomach, respectively. (0.07 MB TIF) [file pone.0014540.s005.tif]
